# Evaluating the Feasibility and Acceptability of a Community-Based, Co-Created Yoga Program for Women with Gynecologic Cancer: A Series N-of-1 Feasibility Study

**DOI:** 10.3390/curroncol32070368

**Published:** 2025-06-24

**Authors:** Jenson Price, Brooklyn Westlake, Jennifer Brunet

**Affiliations:** 1 School of Human Kinetics, University of Ottawa, Ottawa, ON K1N 6N5, Canada; jenson.price@queensu.ca (J.P.);; 2 Cancer Therapeutic Program, Ottawa Hospital Research Institute, The Ottawa Hospital, Ottawa, ON K1H 8L6, Canada; 3 Institut du Savoir Montfort, Hôpital Montfort, Ottawa, ON K1K 0T2, Canada

**Keywords:** mind–body, oncology, single-case, community

## Abstract

Purpose: Current yoga programs for cancer survivors do not meet participants’ needs and are rarely implemented in community-based settings, despite reported benefits. The aim of the current study was to implement a co-created 12-week bi-modal Hatha-based yoga program for adults diagnosed with gynecologic cancer in the community and assess the feasibility and acceptability of the program and study methods. Methods: Using a mixed methods series N-of-1 A_1_BA_2_ research design, participants were recruited from The Ottawa Hospital. Participants self-selected a morning or evening program, completed surveys 9 to 11 times and were interviewed post-program. The yoga instructor was interviewed post-program about her experience delivering the program. Quantitative feasibility outcomes were tracked throughout the study. Qualitative acceptability outcomes were explored during post-program semi-structured interviews. Audio and video recordings of the yoga classes and data from the instructor interview were used to assess fidelity outcomes to determine whether the protocol could be adhered to consistently. Results: Forty-one individuals were screened for eligibility and 20 consented (48.7%). Seventeen participants (85.0%) completed the final survey. Participants attended 83.1% (19.9/24) of classes with varied engagement with optional features. The instructor was 61.3% adherent to the prescribed protocol, using recommended behaviors 44.6% of the time. Participants shared barriers and facilitators that influenced the success of the trial methods and program. Conclusions: The program was well-received and trial methods were moderately successful, but refinements are warranted before a large-scale trial. Community-based yoga programs could be feasible and acceptable for women with gynecologic cancer.

## Simple Summary

Yoga could be beneficial for addressing the side effects of gynecologic cancer, but most yoga programs are not designed specifically for the needs of women with gynecologic cancer. This study tested a 12-week yoga program that was created with women with gynecologic cancer and yoga instructors to determine the feasibility and acceptability of the program and study methods. Twenty women joined the program and chose either a morning or evening class. Most attended regularly and completed the study with the instructor following the planned program most of the time. Feedback from participants and the instructor helped identify what worked well and what could be improved. The program was generally well-liked, and the study methods mostly worked, suggesting that offering community-based yoga for women with gynecologic cancer is both possible and promising after some minor changes.

## 1. Background

Each year, over 110,000 women in Canada receive a cancer diagnosis, with gynecologic cancers—including ovarian, endometrial/uterine, cervical, vulvar, and vaginal cancers—representing more than 11% of these cases [1]. Commonly reported side effects include both physical (e.g., fatigue, scarring, pain; [2,3]) and psychological outcomes (e.g., stress, fear, negative self-perception; [4,5]), all of which substantially diminish quality of life—a multi-dimensional construct that integrates physical, psychological, emotional, cognitive, and social domains [4,6]. Although traditional definitions of quality of life do not always explicitly include sexual health, this domain is crucial for individuals diagnosed with gynecologic cancers, with the majority reporting decreased sexual activity [7,8] and increased sexual concerns post-treatment [2,9]. Given the persistent physical and psychological challenges that women diagnosed with gynecologic cancer face, supportive care services are vital to improving quality of life.

Psychosocial and lifestyle supports, while essential, are often underprovided within the healthcare system [10,11]. Physical activity has been widely recognized for its role in mitigating adverse physical and psychosocial side effects of cancer and its treatments, reducing comorbidities and secondary malignancies, and enhancing patient-reported outcomes such as cardiovascular health, muscle strength, fatigue, mood, self-esteem, anxiety, and depression [12,13]. Among various physical activities, yoga has emerged as uniquely beneficial due to its holistic approach, integrating physical postures, breathing exercises, meditation, and mindfulness techniques [14,15]. Yoga specifically fosters a mind–body connection, enabling participants to positively address emotional, cognitive, and behavioural challenges associated with cancer-related physical and psychological changes [16]. Empirical evidence suggests yoga’s potential to enhance quality of life, alleviate depression and anxiety, reduce fatigue, and improve sleep among breast cancer survivors [14,17,18]. Although the evidence regarding yoga’s effects on body image and sexual health remains mixed, yoga’s distinctive holistic approach and emphasis on mindfulness and self-awareness position it as particularly suited to addressing these concerns [9,19,20,21]. However, a meta-synthesis revealed a mismatch between women’s needs post-cancer diagnosis and existing yoga programs [22]; this highlights the necessity of involving end-users in developing tailored yoga programs that address specific psychosocial needs and preferences to increase program accessibility and effectiveness.

In 2021, an interdisciplinary partnership was formed to develop a community-based yoga program for adults diagnosed with gynecologic cancer [23]. The collaboration involved researchers from the University of Ottawa, a psychologist from The Ottawa Hospital, a representative from the Ottawa Regional Cancer Foundation (ORCF), and intended end-users (i.e., adults diagnosed with gynecologic cancer and yoga instructors). Guided by the Knowledge-to-Action (KTA) framework [24,25], the co-creation process prioritized end-users’ lived experiences and needs, resulting in a 12-week bi-modal program featuring two weekly 60 min Hatha yoga classes for groups of five–seven participants, with optional features including organized group discussions, journalling, and pre-recorded videos for at-home practice [23]. Hatha yoga was chosen for its emphasis on physical asanas and breath control, which can improve physical symptoms and reduce fatigue [26]. Group-based classes were chosen with the aim of enhancing social support [22], a critical factor in improving the quality of life among adults diagnosed with cancer [27], especially when group members share similar features (such as cancer type; [22,28]). The bi-modal format is intended to combine online accessibility with in-person support, addressing barriers such as geographical distance while providing a supportive environment [28]. As for the optional features, organized group discussions and journalling were integrated as they can bolster psychosocial wellbeing and self-efficacy [28,29,30,31], with journalling promoting emotional processing and reducing stress and anxiety [30,31]. Last, pre-recorded videos were available to offer additional practice opportunities, fostering a sense of routine and self-discipline [28].

Before conducting a definitive trial to assess the program’s direct and indirect effects, it is essential to first establish the operational viability of trial methods and program features (i.e., feasibility), alignment with end-users’ values and preferences (i.e., acceptability), and consistency between the intended design and actual execution (i.e., fidelity) [32]. The process of evaluating the implementation of a community-based program is integral to ensure that it closely aligns with the specific needs of the target population, thereby maximizing resource efficiency and effectiveness [33,34]. This not only ensures the program’s relevance and efficacy but also contributes significantly to the body of scholarly research, guiding future programs and policy decisions [33,34]. Building on the previous co-creation process [23], this study addressed the next two phases in the KTA framework [25]: implementing the program (phase 4) and monitoring the implementation of the program (phase 5). The objectives were to: (1) assess the trial methods feasibility and acceptability, (2) assess the program feasibility and acceptability, and (3) assess the program protocol fidelity and acceptability. An additional objective was to explore the perceived benefits for participants, as they may influence acceptance and willingness to participate.

## 2. Methods/Design

This study utilized a series N-of-1 concurrent triangulation design, wherein quantitative and qualitative data were collected and analysed concurrently [35]. The quantitative feasibility and fidelity data offers insight into the efficiency and practicality of the study methods and program to better guide future resource allocation and timelines, and the qualitative acceptability data complements these metrics by delving into end-users’ perspectives, uncovering barriers and perceived benefits, facilitating improvements, and ensuring the study methods and program align with their lived experiences and needs. This manuscript adheres to the Consolidated Standards of Reporting Trials 2010 statement for N-of-1 trials [36] and the 21-item Checklist Standardising the Reporting of Interventions For Yoga guidelines [37]. The study was registered on ClinicalTrials.gov (no.: NCT05610982) on 3 November 2022; detailed methods are available in the published protocol [38].

### 2.1. Procedures

Ethics approval was granted by the Ottawa Health Science Network Research Ethics Board (file no.: 20220544-01H) and by the University of Ottawa’s Office of Research Ethics and Integrity (file no.: H-10-22-8671). The trial employed a repeated baseline approach with a follow-up phase, an A_1_BA_2_ design [39,40,41]. After providing informed consent and self-selected participation in the morning or evening program, participants were randomized (1:1:1) to baseline phase durations (3, 4, or 5 weeks) using a computer-generated sequence created via the National Cancer Institute Clinical Trial Randomization Tool managed by JP. As this randomization applied only to the baseline phase (A_1_), traditional allocation concealment procedures were not applicable as participants would know when they were completing the surveys. Participants completed weekly online surveys during the baseline phase (A_1_), then again at 3 time points during the 12-week yoga program (B), and at 3 time points during the 8-week follow-up (A_2_). Participants were asked to complete surveys within 48 h of receiving the link. Two weeks after the program, participants and the instructor completed a semi-structured interview via Microsoft Teams. Table 1 presents the schedule of assessments.

### 2.2. Participants

Adults diagnosed with stages I–III gynecologic cancer were recruited via healthcare provider referral, registry mailout of invitation letters, posters, and word of mouth. Inclusion criteria were age ≥ 18 years, English proficiency, Internet access, and willingness to travel to the ORCF week 1 and 2 of the yoga program. Exclusion criteria included being non-ambulatory or regularly practicing yoga (≥1 session/week over the past 6 months), as these factors could compromise participant and instructor safety during physical transitions required in the program and introduce bias in patient-reported outcomes assessed in a forthcoming manuscript, respectively.

### 2.3. Sample Size

A conventional power calculation was not appropriate given the feasibility aims of this study [42] and the novel nature of delivering the program at the ORCF [38]. Instead, a target sample size of 20 participants was selected based on end-user recommendations and pragmatic considerations. During the co-creation process, end-users recommended limiting the class size to 7 participants per program to support individualized instruction [23]. To account for an expected 30% dropout rate based on previous literature [43], the cap was increased to 10 per program. Two concurrent program sessions (morning and evening) were offered to accommodate varying schedules. Given the use of a series N-of-1 design, where typical samples range from 1 to 13 participants [40], and the collection of repeated measures, this sample size was sufficient to assess feasibility outcomes via descriptive statistics and support preliminary statistical analyses of individual trajectories using hierarchical linear modeling (reported in forthcoming manuscript).

### 2.4. Yoga Program and Study Setting

Details of the yoga program [38] and the co-creation process [23] are presented elsewhere. The 12-week bi-modal program offered 2 weekly 60 min Hatha group classes at the ORCF. Two weeks before the program, the yoga instructor emailed participants a 1-page intake form and had a 15 min 1-on-1 Zoom consultation to discuss potential concerns. At the first class, each participant received a yoga mat for the duration of the program. Weeks 1–2 were in-person only, weeks 3–12 were offered in-person and via Zoom (password-protected). Classes followed a consistent structure: arrival, warm-up with breath practice, 2 flow sequences, restoration with meditation, optional group discussion, and departure. The instructor tailored the protocol based on intake meetings and ongoing observations, offering modifications using chairs, blocks, or alternative asanas. Participants were encouraged to modify practices themselves, ask for help, rest as needed, and/or visualize practices instead of physical execution based on how they felt. Participants had the option to engage in group discussions after each class, were provided a journal during the first class, and had access to an online database of pre-recorded practices starting week 3.

### 2.5. Training

A certified yoga instructor with 10+ years of experience working with clinical populations and pursuing a yoga therapist certification was hired to deliver the morning and evening programs. Two Human Kinetics undergraduate students assisted during classes (1 per program). All team members received a half-day training session led by JP, which covered gynecologic cancer treatment and side effects, the program structure, intended instructional behaviors, safety protocols, and adaptive strategies. The instructor received the Instructor Guidebook (Version 2; [23]), containing detailed session content, recommended behaviors, and guidance for supporting participant needs. To support consistency and adherence, the instructor kept detailed notes after each class documenting what was delivered, any adaptations made, and participant responses. Standardization and support were maintained through biweekly 30 min team meetings to provide ongoing guidance and discuss participants’ progress, challenges, tailoring efforts, and class notes as well as the yoga instructor and volunteers’ experiences. Participants’ engagement and adverse events (if any) were reviewed to ensure safety.

### 2.6. Measures

#### 2.6.1. Feasibility of Trial Methods and Program

To assess feasibility, recruitment, retention, adherence, and engagement rates were tracked. A priori benchmarks indicating success were set based on relevant literature from yoga intervention trials [28,44,45,46], methodological guidance for feasibility studies [42], and pragmatic considerations related to the study context. The recruitment rate was defined as the proportion of screened adults who consented to participate; a rate of ≥50% was considered successful based on previous yoga intervention trials, which typically report low recruitment rates (e.g., <40%; [44]). The retention rate was defined as the proportion of enrolled participants who completed the final survey at week 20 (i.e., 8-weeks post-program); a rate of ≥75% was considered successful, consistent with established dropout rate thresholds [47], which is pertinent for future large-scale trials [42]. Program adherence was defined as the proportion of classes attended out of 24, regardless of modality; adherence was successful if participants attended ≥75% of classes, aligning with rates observed in other yoga trials [45,46] and the recommendations of the co-creation participants [23]. Engagement included attendance and participation in group discussions, self-reported completion of journal entries, and self-reported viewing of pre-recorded practices. Group discussion engagement was considered successful if ≥50% of participants attended each optional group discussion and ≥50% of those attendees spoke at least once, which was considered indicative of meaningful engagement via regular and active interaction among participants [48]. Journal engagement was considered successful if ≥50% completed ≥1 journal entry a week and pre-recorded video engagement was considered successful if ≥50% watched ≥1 pre-recorded video a week during ≥50% of the accessible period (5 weeks). These thresholds were selected to reflect moderate engagement expectations that are achievable but meaningful in the context of low-burden, self-directed components of yoga programs [28].

#### 2.6.2. Acceptability of Trial Methods and Program

Participants were asked to take part in a semi-structured interview to discuss their opinions regarding the trial methods and program. Interviews were audio-recorded, conducted by JP, and guided by an interview guide with open-ended questions addressing the following: suitability of trial methods, reasons for enrolling, facilitators or deterrents for completing measures, the program’s overall relevance, benefits, suitability, and problems/concerns experienced during the trial and/or program.

#### 2.6.3. Fidelity to and Acceptability of Program Protocol

Class recordings were coded to assess adherence to the prescribed structure and instructor behaviors. Fidelity was confirmed if ≥75% of class phases were the prescribed length and if ≥75% of the instructor’s behaviors matched the recommended behaviors outlined in the Instructor Guidebook. During the audio-recorded interview with the instructor conducted by JP, she was asked about her experience delivering the program, thoughts on program content and the Instructor Guidebook, delivery and content of training, ongoing supervision, any difficulties encountered, potential amendments, and views on fidelity to the program protocol.

#### 2.6.4. Additional Measures

Personal and medical factors were collected via self-report in the first survey only. Sociodemographic information included age, gender identity, ethnicity, civil status, work/education status, household income, and comorbidities. Medical information included height, weight, cancer type and stage, treatments received, and self-reported physical and mental health on a 5-point scale (adapted from the RAND-36; [49]). At each time point, participants were asked to complete an online survey comprising quality of life, perceived cognitive abilities, fatigue, sexual distress, negative body image, and perceived stress; findings will be reported in a forthcoming manuscript.

### 2.7. Data Analysis

Descriptive statistics were calculated for quantitative feasibility outcomes in Microsoft Excel. For each metric (i.e., recruitment, retention, adherence, and engagement), proportions and percentages were calculated by dividing the number of participants meeting the specific criterion by the total number relevant to that metric (see Section 2.6.1 for details). For example, one participant who withdrew during week 2 of the program and did not attend any further classes was included in the denominator when calculating recruitment and retention rates but was excluded from the adherence and engagement rates, which only considered participants who remained enrolled during the intervention period.

Manifest content analysis was used to analyze the qualitative data, which focuses on identifying and organizing explicit content within participant responses [50]. Interviews were transcribed verbatim and imported into in NVivo for coding and organization. An initial coding framework was developed by JP based on the interview guide and research objectives. JP then deductively coded the transcripts line-by-line, with openness to inductive additions as relevant ideas emerged. Codes were iteratively grouped into categories and higher-order themes through constant comparison, and summary tables were developed to synthesize key findings, with JB acting as a critical friend throughout the process.

Instructor fidelity to the protocol and the use of recommended behaviors were assessed using a duration and frequency analysis of all class recordings. The coding scheme for both class structure and instructor behaviors was developed by JP based on the class protocol and listed behaviors in the Instructor Guidebook, which were established during the co-creation process [23]. Class structure was coded according to the six prescribed segments of the yoga class structure (i.e., arrival, warm-up, flow sequence 1, flow sequence 2, restoration, and departure). Fidelity to class structure was assessed based on whether each segment met the target duration within a ±30% threshold. Instructor behaviors were deductively coded using a predefined scheme, whereby coders watched each video and recorded the number of times each specified behavior occurred. These included recommended (e.g., invitational language, prompting introspection, offering choices) and non-recommended (e.g., dictating language, use of qualifiers) behaviors. The coding scheme also included an “emergent behavior” category to capture unanticipated but recurring behaviors. Two undergraduate students (BW, MP) were trained and supervised by JP; they practiced coding class structure and instructor behaviors together in the first 4 classes to achieve consistency. Then, they independently coded another 8 classes and interrater reliability was calculated using Cohen’s Kappa (κ); agreement was defined as both coders identifying the same behavior at the same time point. The resulting κ was 0.72, indicating substantial agreement based on established benchmarks [51]. After achieving this threshold, BW coded the remaining 36 classes independently.

Data were collectively interpreted using a mixed-methods convergence matrix to assess convergence and/or dissonance.

## 3. Results

Table 2 summarizes participants (N = 20) sociodemographic and medical characteristics. Below, the quantitative results are summarized with a brief overview of the corresponding qualitative findings. Table 3 provides a detailed breakdown of the feasibility metrics with a comparison between the two programs, Table 4 provides an overview of the qualitative findings, and Supplemental File S1 presents the supporting quotations for the qualitative findings (Table S1).

### 3.1. Recruitment Rate

**Figure 1:**
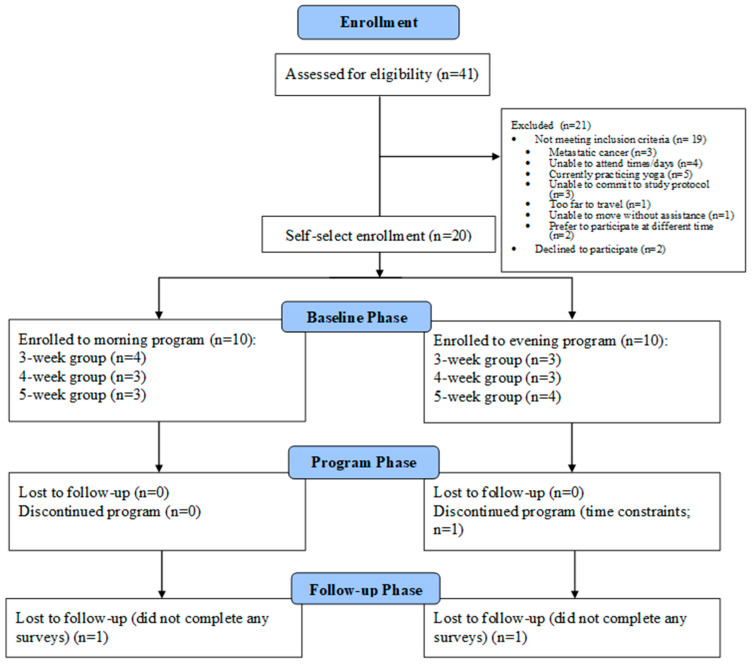
CONSORT 2010 Flow Diagram.

Potential participants were identified via only the registry mailout of invitation letters. Out of 500 mailed invitation letters, 55 individuals (11% response rate) contacted the research team within 3 weeks. The screening rate was 74.5% (41/55); 2 could not be reached after 3 contact attempts and 12 were not screened for eligibility because the target sample was reached (see Figure 1 for participant flow). Participants reported being surprised but appreciative and excited when they received the invitation to participate. Their reasons for wanting to participate in the *trial/research* included: giving back and contributing to future women’s experiences, finding strategies to cope with side effects, (re)starting yoga, and using the program as an opportunity to test out a new lifestyle, either by increasing their physical activity or working less.

The eligibility rate was 53.6% (22/41; see Figure 1 for exclusion reasons). Two declined participation because they were >10 years post-treatment and felt the program would be more beneficial to someone closer to treatment while the remaining 20 (90.9%) consented to participate, yielding a recruitment rate of 48.8% (20/41). After the first 14 eligible participants self-selected morning or evening sessions—filling the morning program to capacity—the remaining 6 were informed only about the evening program; unprompted, only 1 expressed preference for a morning program. For participants, their reasons for wanting to participate in the *program* included: in-person, group sessions with similar others to reduce pandemic-related isolation, the program’s winter timing, and the location of the ORCF.

### 3.2. Retention Rate

The retention rate was 85.0% (17/20) for completing the final online survey. No adverse events or unintended consequences were reported by participants or observed by the research team during the study. One evening participant withdrew from the trial during week 2 of the program (after class 3) and did not complete any Phase B or A_2_ surveys. The other two participants completed the full 12-week program but did not complete any follow-up (A_2_) surveys. In addition, 18 participants (90.5%) completed the post-program interview (morning = 9/10, evening = 9/10). Participants identified two main motivators (sense of obligation and altruism) and one facilitator (online survey and interview platforms) for completing data collection tasks. Three main deterrents for completing the surveys mentioned were: struggles with questions pertaining to certain outcomes (i.e., general quality of life, female sexual distress), timeframe for questions (i.e., last 7 days), and question redundancy.

### 3.3. Adherence Rate

The adherence rate was 83.1% (19.9/24) and 89.4% (17/19) of participants attended >75% of classes. In-person attendance (56.5%) was higher than online attendance (34.2%). Participants noted five main elements as fundamental to wanting to attend classes: (1) ideal amount of yoga congruent with lifestyle and needs, (2) in-person and group-based facilitated connection with peers, (3) mandatory in-person attendance for the first 2 weeks set foundation and connections to build upon, (4) knowledgeable and supportive instructor with a competent assistant, and (5) content that supported them to connect with their bodies and supported their perceived wellbeing. The two participants who attended ≤75% of classes reported illness as the primary reason for not attending class; however, other participants shared that family and work commitments sometimes conflicted with their participation.

### 3.4. Optional Program Feature Engagement Rate

Engagement with optional program features varied. *Pre-recorded videos* were the least utilized; only 5 participants (26.3%) viewed them, totaling 11 views (range = 0–4). Participants felt the live classes met their needs and suggested that recordings of the actual classes might be more beneficial. *Journalling* had limited engagement, with only 15.7% (3/19) of participants writing ≥1 journal entry a week (range: 0–24 entries, median = 5, Q1; Q3 = 3.25; 9.5). Participants held different views of the journals; some sought to journal to remember their thoughts and class content, some avoided journalling in favor of processing experiences internally, and others forgot about the journals when they stopped receiving prompts to use them from the instructor. *Group discussions* was the most used optional feature. Overall, 91.6% (44/48) of group discussions were attended by ≥50% of participants with 79.4% (35/44) of attendees speaking at least once per discussion. As noted above, the connection participants formed with each other was a motivating factor for attending the classes. Participants further shared that the group discussions allowed for progressive connections that grew with the continuous sharing of similar experiences, which facilitated emotional comfort and processing of shared experiences. Participants found it difficult to connect with those who attended mostly online and vice versa. While some participants were disappointed about the lack of connection online, participants who attended primarily online acknowledged that the convenience outweighed the lack of connection.

### 3.5. Fidelity to Class Structure

See Table 5 for a summary of fidelity to class structure. Overall, class structure fidelity was 61.3% (162/264). The instructor adhered to the protocol for the evening program (62.5%) slightly more than the morning program (60.1%), which was delivered prior to the evening program. The class segment with the highest fidelity was *sequence 2* with the instructor adhering 91.7% for both the morning and evening program. There were notable deviations from the prescribed protocol for four class segments. In both programs, the *warm-up* consistently exceeded the prescribed duration because the instructor used this time to provide education on body anatomy and how yoga improves bodily function. The time spent on *sequence 1* steadily decreased in both programs, attributed to participants gaining familiarity with the sequence, requiring less explanation and executing it faster. In classes 1–7, sequence 1 lasted at least the prescribed 10 min in 57.1% of morning classes and 42.9% of evening classes. However, in classes 8–24, the duration was below the prescribed 10 min in 100% of classes for both programs. The time allocated to *group discussions* varied between the two programs; during the last half of the program (classes 12–24), the morning program met or exceeded the prescribed 10 min in 75% (9/12) of classes, while the evening program only did for 16.7% (2/12) of classes. Finally, the *departure* segment was not consistently recorded, with recordings being stopped when the instructor thanked participants, omitting any post-class interactions. Therefore, when calculating program fidelity without the departure component, the overall fidelity was 66.0%, with the evening program at 66.7% and the morning program at 65.3%

### 3.6. Fidelity to Recommended Behaviors

Table 6 provides the list of recommended and non-recommended behaviors, along with fidelity data. Overall, instructor fidelity was 44.6%; the most used recommended behavior was prompting introspection (20.7%) and the most used non-recommended behavior was dictating language (42.6%). The instructor clarified the use of dictating language during her interview; she would cue them through several variations using dictating language and then encouraged them to introspect and take ownership of their practice by connecting with their bodies and choosing the most appropriate movement for that moment. This was done to ensure participants were aware of their options for movement given the “beginner” nature of participants’ experience with yoga. In addition, the instructor regularly engaged (9.5%) in sharing her personal experience practicing yoga, which was a behavior captured in the “emergent behaviors” section of the codebook. Interestingly, participants appreciated this behavior. The instructor’s age (middle age) and gender (woman) were noted as beneficial by participants because they identified with her struggle in asanas (e.g., losing balance), creating a sense of safety and comfort.

#### Instructor Acceptability

Supplemental File S1, Table S2 provides quotations supporting the following findings. The instructor provided valuable insights into the yoga program’s beneficial aspects. Comprehensive training and a well-organized Instructor Guidebook boosted her confidence and streamlined class structure and timings. Intake meetings and forms helped tailor content to participants’ physical abilities and concerns. The modifiable protocol allowed for autonomy and kept classes engaging. Appropriate class length and frequency fostered connections amongst participants, group cohesion, and noticeable progress. The volunteer assistant was essential, enabling the instructor to focus on teaching rather than logistical tasks like equipment setup and technical troubleshooting.

However, challenges included the class size limit of 10 (as the dropout rate during the trial was below the projected 30%), which constrained her ability to give individual attention. She suggested formal check-ins or enhanced technical setups like larger screens for improved facilitation for participants online. She suggested incorporating additional content in the Instructor Guidebook for a more holistic approach, such as breath awareness practices, instructor preparation guidance, class themes, and pelvic floor education. Additional features that could enhance the program also included a longer shavasana, an optional curated music playlist, multidirectional microphones for group discussions, and a peer leader to ease the instructor’s burden and enhance dialogs.

## 4. Discussion

This study demonstrated the feasibility and acceptability of a co-created, community-based 12-week bi-modal Hatha yoga program for adults diagnosed with gynecologic cancer. Retention, adherence, and group discussion engagement exceeded targets, while recruitment, journaling, and at-home video use warrant further refinement. Participants responded positively to the program’s dosage, modality, delivery, social connection, and impact on perceived wellbeing. The bi-modal delivery was unique to this program and population [52,53,54] and highly valued, providing flexibility and accommodating participants’ needs. Participants provided valuable insight into the strengths and pitfalls of certain trial methods and program features, allowing for meaningful refinement.

For recruitment, nearly 50% of individuals screened were ineligible and 21.8% were not screened because the programs reached maximum capacity. While the sheer number of individuals who made contact with the research team indicates interest in the program, a surplus of ineligible or unscreened people is burdensome for both potential participants and researchers [55]. To address this, recruitment materials could be refined with end-user input to ensure eligibility criteria clarity [56], more programs could be run concurrently to accommodate interest, and recruitment efforts could be optimized to balance response rates and resources. For retention, participants were asked to complete 9 commonly used measures via online surveys 9, 10 or 11 times (depending on baseline randomization), achieving a final retention rate of 85.0%, notably higher than many physical activity interventions targeted at adults diagnosed with cancer [57]. Importantly, participants reported completing surveys out of obligation or altruism and expressed dislike for them, but they did not raise concerns about the number of time points. Still, their externally driven motives bring into question the generalizability of the retention rates. Being an older adult, a woman, and having certain psychosocial traits (e.g., empathy) predicts altruistic behavior [58,59,60], which may partly explain the current retention rate. Refining the surveys to address concerns raised (e.g., shorten and include open-ended questions) and keeping it online may enhance completion rates. That being said, identifying alternative means of motivating data collection completion that does not rely on empathy or guilt will be imperative for future trials.

Adherence rates were high, and acceptability data suggest participants perceived benefits to their wellbeing and quality of life by focusing on their physical and mental selves during classes. Group discussions, which also had high engagement, benefited participants by facilitating emotional comfort and processing of shared experiences, which underscores the importance of retaining this optional feature. Conversely, journalling did not meet the engagement thresholds, warranting reconsideration of its relevance. A size-inclusive yoga and body gratitude journal study [61] found 71% of participants journalled ≥1–2 times a week compared to 15.7% in this study. The difference may be due to the “mandatory” journalling in the former study or perceived lack of benefit reported herein. Similarly, at-home video use was low. The success of at-home video use in yoga interventions varies [28,62]. Participants suggested providing class recordings instead and reminders to increase engagement. Moving forward, considering some valued these offered features, they could remain as optional to allow future participants to tailor their experience to avoid artificially inflating engagement by requiring features that may not be meaningful or beneficial for all [63]. Future iterations should explore participants’ preferences for accessing and receiving reminders about optional features, possibly through automated emails.

Program fidelity, particularly the instructor’s adherence to class structure, was moderate. The instructor deviated from the protocol to provide education, a behavior participants found beneficial. This suggests that an adaptive approach to fidelity, prioritizing participants’ benefit over strict adherence, may be valuable. This resonates with debates in exercise psychology regarding the balance between fidelity and adaptability [64]. In addition, results revealed additional beneficial behaviors (e.g., instructor sharing personal experiences), which helped participants feel safe and confident. Nonetheless, as each groups’ needs may differ, gathering regular feedback from participants on what behaviors are beneficial could offer instructors insights into the impact of their teaching style and content adaptability, helping them tailor their approach to better meet participants’ needs and potentially enhance program effectiveness.

This study extended previous work [23] informed by the KTA framework by implementing the program (phase 4) and monitoring the implementation of the program (phase 5). Monitoring implementation outcomes [65] helps determine if a program’s success (or lack thereof) is due to program content or the implementation process and provides helpful insights into the future viability of the program [66,67]. In addition to the implementation metrics reported herein, additional implementation findings were anecdotally observed by the research team. First, in terms of penetration, the partnership between The Ottawa Hospital for recruitment and the ORCF for program delivery facilitated access to support services that participants were unaware of, addressing a reported gap in survivorship care [68]. Second, while the current program was offered in the community at no charge due to a research grant, in-kind contributions from the ORCF, donations, and student volunteers, it is estimated to have cost close to $18,000 (e.g., staff and instructor salary, yoga equipment, room rental) to deliver the program. However, the recurrent costs for an organization with the requisite space would mainly be the instructor’s salary. Participants expressed their willingness to pay for the program, increasing the likelihood of long-term implementation. Several hybrid models of funding could be explored to support sustained implementation, including a mix of fee-for-service (upfront, set cost per session) and subsidized costs (total session costs off-set through donations, sponsorship) [57]. Future research will need to assess effectiveness and continue evaluating implementation outcomes to ensure sustainability.

## 5. Limitations

The limitations of this study should be acknowledged. First, it was a relatively homogeneous sample; thus, findings may not generalize to younger or older women or other ethnocultural groups. Second, the morning and evening programs were delivered by the same instructor; therefore, a future study is needed to assess if the acceptability of the intervention and fidelity is the same across instructors to determine if specific instructor factors are important. Third, two participants did not participate in the interview, so their perspectives are unknown. Fourth, while this feasibility study was not designed to assess broader generalizability or adjust for confounders such as baseline physical activity or mental health, these considerations should be addressed in future trials focused on effectiveness and scalability.

## 6. Conclusions

The results from this study support the feasibility and acceptability of a co-created, community-based 12-week bi-modal Hatha yoga program for adults diagnosed with gynecologic cancer. Participants appreciated the flexibility, relevance, and sense of community it fostered. The instructor’s ability to connect with participants through shared experiences and personalized instruction emphasized the critical role of instructor characteristics in the program’s success. Moving forward, refinements are needed to enhance recruitment strategies, optimize the use of optional program features, and ensure the program’s sustainability in ‘real-world’ settings. This work contributes to the growing body of evidence supporting the benefits of mind–body interventions in oncology settings and emphasizes the significance of involving end-users in the development and evaluation of health interventions to ensure their relevance, effectiveness, and sustainability.

## Figures and Tables

**Table 1:** Schedule of assessments for gynecologic cancer yoga program.

Phase	Time Point (s)	Date (s)	Survey Package	Participant Interview	Instructor Interview
Recruitment Phase	−9 to −7 Week	28 November–22 December 2022			
Baseline Randomization	−6 Week	23 December 2022			
Baseline A_1_ Phase	−5 Week	26 December 2022	X *		
	−4 Week	2 January 2023	X *		
	−3 Week	9 January 2023	X		
	−2 Week	16 January 2023	X		
	−1 Week	23 January 2023	X		
Program B Phase	1 Week (class 1)	31 January 2023	X		
	6 Week (class 12)	9 March 2023	X		
	12 Week (class 24)	20 April 2023	X		
Follow-Up A_2_ Phase	+1 Week	27 April 2023	X		
	+2 Week	4 May 2023		X	X
	+4 Week	18 May 2023	X		
	+8 Week	15 June 2023	X		

Notes. * = depending on allocation.

**Table 2:** Summary of participants’ characteristics.

	Combined		Morning		Evening	
	n	(%)	n	(%)	n	(%)
Age (mean years ± SD, range)	62.4 ± 11.7, 34–80		69.8 ± 6.1, 60–80		55 ± 11.5, 34–74	
Civil status						
Married	14	(70)	7	(70)	7	(70)
In a relationship	2	(10)	1	(10)	1	(10)
Widowed	2	(10)	2	(20)	0	(0)
Never married	2	(10)	0	(0)	2	(20)
Education						
Completed Graduate School	4	(20)	2	(20)	2	(20)
Completed University/College	11	(55)	4	(40)	7	(70)
Some of University/College	3	(15)	2	(20)	1	(10)
Completed High School	2	(10)	2	(20)	0	(0)
Annual household income ($CAD) ^a^ (mean ± SD, range)	112,909.10 ± 73,831.5, 12,000–300,000		107,428.5 ± 89,821.7, 12,000–300,000		122,500 ± 42,914.6, 85,000–180,000	
Employment status						
Full-time	7	(35)	3	(30)	4	(40)
Part-time	4	(20)	1	(10)	3	(30)
Retired	9	(45)	6	(60)	3	(30)
Have children	16	(80)	10	(100)	6	(60)
Age of children ^b^ (mean ± SD, range)	32.7 ± 13.05, <1–50 years		37.3 ± 11.3, <1–50 years		24.31 ± 12.1, <1–38 years	
Comorbid conditions						
Diabetes	4	(20)	1	(10)	3	(30)
High blood pressure	7	(35)	4	(40)	3	(30)
High cholesterol	6	(30)	2	(20)	4	(40)
Ethnocultural background ^c^*						
White	16	(80)	8	(80)	8	(80)
White and South Asian	2	(10)	1	(10)	1	(10)
Latin American	1	(5)	1	(10)	0	(0)
Time since diagnosis ^c^ (mean years + SD, range)	7.6 ± 4.9, 2.7–22.1		7.3 + 6.0, 2.7–22.1		7.9 + 3.4, 3.3–11.3	
Cancer type ^d^						
Ovarian	5	(25)	3	(30)	2	(20)
Cervical	6	(30)	1	(10)	5	(50)
Vaginal	1	(5)	1	(10)	0	(0)
Endometrial/uterine	9	(45)	5	(50)	4	(40)
Cancer stage						
0	1	(5)	1	(10)	0	(0)
I	10	(50)	3	(30)	7	(70)
II	5	(25)	3	(30)	2	(20)
III	3	(15)	2	(20)	1	(10)
Do not know	1	(5)	1	(10)	0	(0)
Treatment type *						
Surgery	17	(85)	8	(80)	9	(90)
Radiation	11	(55)	4	(40)	7	(70)
Chemotherapy	10	(50)	6	(60)	4	(40)
Current hormonal therapy	1	(5)	0	(0)	1	(10)
Changes in weight						
No change in weight	8	(40)	6	(60)	2	(20)
Lost > 11lbs	6	(30)	2	(20)	4	(40)
Gained > 11lbs	6	(30)	2	(20)	4	(40)
Self-rated mental health						
Okay	2	(20)	1	(10)	1	(10)
Good	8	(40)	2	(20)	6	(60)
Very good	5	(25)	3	(30)	2	(20)
Excellent	5	(25)	4	(40)	1	(10)
Self-rated physical health						
Okay	3	(15)	1	(10)	2	(20)
Good	12	(60)	5	(50)	7	(70)
Very good	5	(25)	4	(40)	1	(10)
BMI						
Underweight (<18.9 kg/m^2^)	1	(5)	1	(10)	0	(0)
Normal weight (19–24.9 kg/m^2^)	5	(25)	4	(40)	1	(10)
Overweight (>25 kg/m^2^)	14	(70)	5	(50)	9	(90)

Notes. a = 11, b = 31 children, c = 19, d = 21 (1 participant had 2 primary gynecologic cancers), * = participants could select more than one option.

**Table 3:** Summary of study method and program feasibility metrics.

	Total		Morning		Evening	
	n/N	(%)	n/N	(%)	n/N	(%)
Recruitment						
Response rate	55/500	(11)	N/A	N/A	N/A	N/A
Screening rate	41/55	(74.5)	N/A	N/A	N/A	N/A
Eligibility rate	22/41	(53.6)	N/A	N/A	N/A	N/A
Consent rate	20/22	(90.9)	N/A	N/A	N/A	N/A
Recruitment rate	20/41	(48.8)	10/41	(24.4)	10/41	(24.4)
Retention						
Baseline A_1_ phase	78/80	(97.5)	39/40	(97.5)	39/40	(97.5)
Program B phase	55/60	(91.6)	29/30	(96.7)	26/30	(86.7)
Follow-up A_2_ phase	49/60	(81.6)	26/30	(86.7)	23/30	(76.7)
Post-program interview	18/20	(90)	9/10	(90)	9/10	(90)
Retention rate	17/20	(85)	9/10	(90)	8/10	(80)
Adherence						
Average classes attended	19.9/24	(83.1)	21.8/24	(90.7)	18.1/24	(75.4)
In-person attendance	229/456	(50.2)	129/240	(53.8)	110/216	(50.9)
Online attendance	150/380	(39.5)	78/200	(39)	62/180	(34.4)
Adherence rate	17/19	(89.5)	9/10	(90)	8/9	(88.9)
Program Engagement						
Video engagement rate	0/19	(0)	0/10	(0)	0/9	(0)
Journal engagement rate	3/19	(15.8)	2/10	(20)	1/9	(11.1)
Group discussion attendance rate	44/48	(91.6)	24/24	(100)	20/24	(83.3)
Group discussion participation rate	35/48	(72.9)	19/24	(79.1)	16/24	(66.6)

Notes. N/A = potential participants had not yet self-selected into a program.

**Table 4:** Summary of categories, themes, and subthemes identified in the content analysis of yoga program participants’ (n = 18) post-program semi-structured interview.

Themes	Sub-Themes
Category 1: Recruitment—Reasons for reaching out and enrolling in the study and yoga program	
Community and support	In-person programming post-pandemic
	Group-based programming with similar others
	Giving back to the cancer community
Health and lifestyle improvement	Timing of classes fit their schedule
	Convenient location
	Seeking program to cope with side-effects of cancer
	Start or get back into yoga
	Opportunity to test out a new lifestyle
Category 2: Retention—Factors that may have impacted the completion of data collection measures	
Commitment to contribution and convenience facilitates data collection	Sense of obligation
	Altruism
	Convenience of online surveys
Difficulty navigating content and structure hinders data collection	Struggled with topic area of questionnaires
	Struggled with timeframe of questions
	Struggled with repetition in individual questionnaires
Category 3: Adherence—Why people choose to attend the classes	
Accessibility and flexibility of attending classes	Convenient and hospitable location
	Flexibility of bi-modal delivery
Sense of community and relatability	Similar characteristics amongst peers
	Connection to peers
	An instructor that looked and struggled like them
Personalized and supportive learning environment	First 2 weeks of program in-person to set a foundation
	An instructor that was flexible in what they delivered and incorporated their wants and needs
	An instructional-style that was gentle, compassionate, and non-judgmental
	Content that supported them to connect with their body
Category 4: Program engagement—Value and use of optional program features	
At-home videos were not utilized	No need for more yoga
	Recordings of full classes to support continuity of practice
The journals fit different needs	
Group discussions built connection *in-person*	Group discussions were motivating for attending class
	Group discussions supported progressive connection with peers
	Group discussions supported a sense of community
	Struggle to connect with online peers

**Table 5:** Yoga instructor’s fidelity to class structure.

	Total				Morning				Evening			
	Time		Fidelity		Time		Fidelity		Time		Fidelity	
	Avg Min	SD	n	%	Avg Min	SD	n	%	Avg Min	SD	n	%
Arrival (5 min)	**7.5**	**5.0**	**30**	**62.5**	6.1	2.5	15	62.5	**8.9**	**6.3**	**15**	**62.5**
Warm-up (15 min)	20.0	6.6	25	52.1	**21.6**	**6.0**	**14**	**58.3**	18.4	6.9	11	45.8
Sequence 1 (10 min)	*6.6*	*2.4*	*18*	*37.5*	*6.5*	*2.2*	*10*	*41.7*	*6.7*	*2.5*	*8*	*33.3*
Sequence 2 (15 min)	15.0	3.7	44	91.7	14.5	4.0	22	91.7	15.5	3.5	22	91.7
Restoration (20 min)	16.4	3.9	36	75.0	16.1	3.9	17	70.8	16.8	4.0	19	79.2
Group discussion (5–10 min)	9.1	4.9	37	77.1	11.9	5.0	16	66.7	6.4	2.8	21	87.5
Departure (5 min)	5.3	4.1	16	33.3	**8.2**	**3.7**	**7**	**29.2**	*2.3*	*1.5*	*9*	*37.5*

Notes. The allowable margin of error was ±30%. Italicize text indicates the phase was >30% under the protocol. Bold text indicates the phase was >30% above the protocol.

**Table 6:** Yoga instructor’s fidelity to recommended behaviors.

	Total		Morning		Evening	
	Total	Avg/Class	Total	Avg/Class	Total	Avg/Class
Recommended						
Invitational language	172.5	7.2	147	6.1	198	8.3
Embracing	51	2.1	46	1.9	56	2.3
Choices	78	3.3	89	3.7	67	2.8
Self-compassion	132	5.5	137	5.7	127	5.3
Increase comfort	401	16.7	379	15.8	423	17.6
Introspection	497	20.7	476	19.8	518	21.6
Tailoring	27	1.1	29	1.2	25	1.0
Consent	0.5	0.0	1	0.0	0	0.0
Answering questions	25.5	1.1	31	1.3	20	0.8
Checking in	37.5	1.6	33	1.4	42	1.8
Not Recommended						
Dictating language	1023.5	42.6	1028	42.8	1019	42.5
Qualifiers	250.5	10.4	232	9.7	269	11.2
Extrospection	263.5	11.0	261	10.9	266	11.1
Emergent behavior						
Sharing personal experience	227.5	9.5	247	10.3	208	8.7

## Data Availability

Contact the corresponding author.

## Supplementary Materials

The following supporting information can be downloaded at https://www.mdpi.com/article/10.3390/curroncol32070368/s1, Table S1: Qualitative responses pertaining to acceptability of study methods and program features for co-created 12-week bi-modal hatha yoga program for adults diagnosed with gynecologic cancer (n = 18). Table S2: Instructor (n = 1) qualitative responses pertaining to fidelity for co-created 12-week bi-modal hatha yoga program for adults diagnosed with gynecologic cancer.

## Abbreviations

ORCF	Ottawa Regional Cancer Foundation
KTA	Knowledge to Action (framework)
