# Supplementary material for: Evaluating the Feasibility and Acceptability of a Community-Based, Co-Created Yoga Program for Women with Gynecologic Cancer: A Series N-of-1 Feasibility Study
Source: Curr Oncol. 2025 Jun 24;32(7):368. doi: 10.3390/curroncol32070368 (PMC12293929; doi:10.3390/curroncol32070368)
Supplement: Supplementary file 1 [file curroncol-32-00368-s001.zip › curroncol-3647284-supplementary.pdf]

Table S1. Qualitative responses pertaining to acceptability of study methods and program features for 12-week bi-modal hatha yoga program for adults diagnosed with gynecologic cancer (n=18)	
Sub-theme and description	Example quote with participant pseudonym
**Reasons for reaching out and enrolling in program (qualitative responses related to recruitment)**	
In-person programming<br>Due to the pandemic, participants were interested in opportunities that were in-person because they were tired of online programming.	"I think it's useful, particularly I'd say coming out of the pandemic, to have the group […] I've had enough online stuff, and also, I mean when the pandemic first started I used to try and carry on some fitness classes online and I just get bored. So, I much prefer the in-person" (Mary, morning program)
Group-based programming with similar others<br>Similarly, participants wanted to alleviate some of the isolation that they felt as a result of the pandemic and considered the program appealing because it was a group of similar others.	"to get out and to meet other women in the same sort of bracket, who you know just recovered or have recovered over the years from chemo and radiation and whatnot, and of a similar age" (Linda, morning program)<br><br>"It was, for me, saving my mental health as well. Yeah, because we were just coming out of the pandemic and my body was terrible for sitting for three years and not doing anything. So, for me this whole program gave me hope. I was extremely excited." (Carol, evening program)
Timing of classes<br>From a practical standpoint, the timing of the classes fit into their schedules (either in the morning for those retired or after work for those still employed).	"Personally, I prefer doing it in the morning because then your body is more apt to wake up as towards the end of the day you tend to be a little bit more tired. For me, I always prefer doing my running or yoga in the first thing in the morning when it's cooler and then you have the energy for the rest of the day." (Deborah, morning program)<br><br>"It fit well. Yes, it worked well in my schedule two nights a week because I can go home after work to have supper and relax before I headed to class." (Brenda, evening program)
Convenient location<br>In addition, some participants commented on the added benefit of the location of the building (although this was not a motivating	"The location was perfect because it's a 10-minute drive for me." (Susan, morning program)

factor for everyone, as several participants noted distance to the building was far).	"I thought it was like super convenient having at the Cancer Center as well too, like it was pretty close to my house, which was great, maybe like a 15-minute drive and free parking, which was great." (Diane, evening program)
Giving back<br>Participants were motivated to participate because they wanted to give back to "science" that had helped them and to also create better supports for future cancer patients.	"It gave me a sense of feeling of giving back, through research. A way of saying thank you maybe and contributing to the experience of future women who are going to have to go through this. So that was definitely that sense of fulfillment that I was doing something bigger than me." (Cynthia, morning program)<br><br>"I realized for me, the reason I participated in the first place, I felt that I wanted to contribute. I said, "Hey, this is a great opportunity contribute, talk about my experiences, especially if they can help other women going through the same kind of treatment and cancer." (Elizabeth, evening program)
Coping with side-effects<br>For some, they were interested in how yoga could help them cope with side-effects from treatment (e.g., sleep, urinary inconsistence).	"I thought it would help more in centralizing some physical issues, like the core strength, I thought that would be more, that in helping bladder and helping more some of the stuff that was damaged through radiation." (Deborah, morning program)
Start or get back into yoga<br>Others had been interested in starting (or getting back into) yoga for a while and jumped at the opportunity.	"for me, you know, my purpose was really I want to get back to doing yoga myself, I want to go through some real guided healing" (Cynthia, morning program)<br><br>"I liked that it really introduced me to yoga because pre-pandemic I did yoga and then nothing since the pandemic. So it motivated me to go back." (Sharon, evening program)
New lifestyle<br>Finally, participants also viewed the program as an opportunity to test out changes they wanted to make in their lifestyle, such as cutting down on the number days they worked.	"I think it for me it went even beyond that, you know, to the extent of testing whether I can do four days a week with work" (Cynthia, morning program)<br><br>"For me, it meant like a good opportunity to get into like a healthy routine." (Diane, evening program)
**Components that may have impacted the completion of data collection measures (qualitative responses related to retention)**	

Sense of obligation<br>Participants acknowledged that they felt they should complete the measures because they were aware of what was asked of them when they signed up for the study.	"If we participated, if we can't fill in forms, do surveys, then we shouldn't bother burdening you with our presence - if we can't cooperate and help in any way we can." (Linda, morning program)
Altruism<br>Participating in research, and thus completing the data collection measures, was considered a necessity to ensure good programming for future cancer survivors or supporting research.	"My daughter had depended on individuals for her research to get her PhD and I thought how can I know that and not agree to participate for you and I was a bit frankly hostile in the beginning in the sense of I don't have time for this." (Barbara, morning program)<br><br>"I enjoy, to me, research is very important and I like to participate in research to help others." (Brenda, evening program)
Completing questionnaires online<br>Of the potential options (i.e., online, via phone, pen and paper), online was considered the best option as it allowed for flexibility of when and where to complete the survey package and time to reflect on answers.	"I liked it on the computer. Yeah. Because then I can go at my own pace, or I could just, you know, go back and forth between questions if I wasn't sure where I was feeling in one particular question, I'd go forward or back, so or just leave it." (Deborah, morning program)<br><br>"Online, totally online, don't give me the pen and paper and don't call me, because then like if I want to take a break and come back. No, no, no online. Easy peasy. Do it on my tablet. I can do it like you know, while I'm, like listening to music or, you know, like petting my cat online. 100%." (Sandra, evening program)
Struggled with topic area<br>Out of the 9 outcomes explored in the survey package, participants predominately raised concerns with the FACT-G, the FSDS, and the SDQ. For the FACT-G and FSDS, participants struggled with the applicability of the questions. While there was the option to answer "never" if the question did not have a barring on their life, they still found the presence of the question unnecessary. In addition, for the FSDS, participants who were	"I often find the you know none of the above or not applicable is something that is just hugely helpful because I know it's a bit of a scapegoat, but I'm a widow and I really find it hard to talk about sexuality. And you didn't allow me an opt out except in the very first question I prefer not to say and it I just I found that so I was very whimsical about it." (Barabara, morning program)<br><br>"Maybe you know as an older person, I hate to say it, but I thought some of the questions were kind of geared to somebody younger than myself, you know? As one gets older, I feel there's a lot more of self-acceptance. […] The questions with regards to the sex. Well, yeah, it's important, but maybe it's, you know, not the biggest issue at my age." (Pamela, evening program)

not sexually active did not believe the questions applied to them, even though the focus of the questions were intrapersonal and not interpersonal. Finally, for the SDQ, participants struggled with understanding "why" of the questions as well as with identifying descriptors for themselves.	
Struggled with timeframe of questions<br>The questions asked participants to reflect on the past 7 days. For some participants, this was difficult when they interpreted the questions as being "truths" (or traits) of who they are and not necessarily changeable (states).	"I did think that it was hard to say in the past week how has this felt, and I think sometimes I would forget that we're only talking about the last week as, you know, how have you felt about this. So, that was a tricky one, and I wouldn't be surprised if I didn't, if I wasn't always consistent on that." (Mary, morning program)<br><br>"Sometimes I found it hard because umm, sometimes like for me, I always have stomach issues or sudden back problems. So when we talk to physical part, I was like, 'OK, is it how I feel now?' So sometimes I had to say no, no, it's true within this last seven days." (Elizabeth, evening program)
Struggled with repetition in individual questionnaires<br>Participants were discouraged by the presence of repetitive questions (i.e., stems that were similar but had slight variation in wording) as they wanted to be consistent in their answering and/or they felt they had already answered the question.	"I think from my experience doing the surveys was not the highlight of my day, but you know, and the fact that they were somewhat repetitive if they had been, you know, different questions, more varied, it would have been more interesting." (Linda, morning program)<br><br>"The surveys were always the same questions, so I kind of repeated myself a lot." (Elizabeth, evening program)
**Why people choose to attend the classes (qualitative responses related to adherence rate)**	
Fit and congruence with lifestyle	
Convenient and hospitable location<br><br>Attending in-person (the preferred method) was facilitated by the location of	"Everybody's so incredibly welcoming, like when you come in, how are you today? How are you feeling today? What are you here for? You know, the yoga program, "ohh yeah, that's great" sign in and then when you go to leave, "how did that work for you? Do you feel well?" I mean, there's just such an aura of support, and it is an

the building and the general welcoming environment that was created by the staff.	outstandingly beautiful building, but the staff. They've just hired the right people to be there.” (Barbara, morning program)<br><br>“That was fabulous, you know? I would drive by there all the time and I never realized, you know what a welcoming place it was and that they offer all these different programs. And so, I liked it and, and the fact that it was smallish, you know, and easy parking and everything was wonderful.” (Pamela, evening program)<br><br>I love the ladies that I greet you as you come in. They are the best, very, very welcoming, full of information and just lovely. It's almost like when you walk in there, I feel like I'm Hoo. Like you know, like it's just somewhere to tell you just walk in and it's like the rest of the shit that you carry with you is kind of gone.” (Carol, evening program)
Flexibility of bi-modal delivery<br>The bi-modal delivery of the program provided participants with the chance to still participate if they had other commitments or were not feeling well enough to attend in-person.	“It was quite a long way from my house, but I didn't mind, you know, getting there, as I said, you were flexible with the zoom for the days where I really couldn't get there or the weather couldn't get me there. So that was not an issue for me.” (Cynthia, morning program)<br><br>“"I did do the beginning couple weeks in person. I did the last class in person but due to the fact that I couldn't fully participate [due to health concerns], I didn't want to kind of you know disrupt the class by me not being able to do it. And then there was a few weeks there where I had to take on more responsibilities at work so it was really difficult for me to get there in time. So the bulk of the bulk of the classes I did remotely.” (Janet, evening program)
Make up of class<br>The timing of the classes (either morning or evening) allowed participants to choose the time that most met their needs, whether that was accommodating for a higher energy in the morning or working during the day. As a result, the sociodemographic characteristics of	“Personally, I prefer doing it in the morning because then your body is more apt to wake up as towards the end of the day you tend to be a little bit more tired. For me, I always prefer doing my running or yoga in the first thing in the morning when it's cooler and then you have the energy for the rest of the day.” (Deborah, morning program)<br><br>“I think like there was a nice mix of people. Like I was one of the younger ones, so like there was me and another girl that were probably closer to 50. And then I know

participants in the groups were more closely aligned (e.g., employment status, age).	that there was some that was that were probably like late 50s. And then there was probably a few in our class that were probably a little bit older. And I know the morning class was like people that were significantly older than us. So I'm glad that's the way that they grouped out, I think it would have been a little bit different if I was in a class with like, you know the 80-year-olds." (Sandra, evening program)
Connection to peers As the program advanced, attending in-person allowed participants to foster and benefit from interacting with similar others through the provision of informal and instrumental support.	"I think I saw us as a group. We had one thing in common to start with, that was gynecological cancer, but we've arranged next Friday, we're all going to go for tea and you know it's sort of we don't want to let it go that we don't want to lose one another. […] To collect another group of friends when you're in your 60s and 70s and maybe some in their 80s, it's not that easy. It's kind of precious and you don't want to throw that away because we all need friends. We all need to have a social life to sort of keep us active and enjoying life, so I think it created a little unit." (Linda, morning program)<br><br>"I like the support of the other ladies around me and they motivate me to actually get it done and do it instead of just saying, "Ohh well, maybe I'll go, maybe I won't". No, I should go get off my ass and go because it is the best thing for my mind, body and soul. So, I loved going in-person and plus you got to see these lovely ladies, and everybody usually had a story or somebody would ask you a question or an opinion or give you some advice." (Carol, evening program)
First 2 weeks of program in-person The first 2 weeks of the program being mandatory in-person set the foundation for connecting with their peers and creating a sense of trust with the instructor.	"You requested we come in person, brilliant, we needed to meet in person because otherwise, I've had to do the zoom connections at times when it just couldn't be avoided for me because I also have a commitment at noon on Thursday so, to get all the way to the general and back wasn't always not the general, but the Cancer Foundation was not always possible and meet that other commitment. But that was crucial that we got together the first two weeks. Huge part of this success, so we could see each other face to face." (Barbara, morning program)<br><br>"I think that it's a good thing in a way because it encourages people to come out right, make it a little bit more of a routine than staying at home." (Sharon, evening program)
Knowledgeable and supportive instructor	

Doing (and struggling) with what she asks them<br>The instructors age and gender were noted as benefits for the participants because they saw themselves in her when she would do the physical postures with the participants and would admit when she was struggling with the postures. This created a sense of safety and comfort.	“Maybe, the fact I think that she was also maybe in a similar age group and maybe you know her own body experiencing certain things she could connect better maybe I don't know you know because obviously I can't compare to another instructor but I felt that as compared to classes that, you know these were private sector classes or, you know, in gyms and things that I try to connect to where you didn't feel the real communication with the instructor. They were just delivering a class and it it's probably my own inhibitions, you know nothing to do with that instructor, but that's what I meant by Jody coming to your level. She gave you time. She was patient and you know, I guess the fact that she works with people in pain. Even though none of us were in our group, were in, you know, in excessive pain, she was used maybe to accommodating people's needs.” (Cynthia, morning program)<br><br>“She was very kind and welcoming and gentle. And also like funny and like realistic as well too. She was like a real person, she was like, ‘yeah, I don't like doing sun salutations on the ground. I use a chair. This is modification I do.’ And I think that kind of gives people some permission, like even if the yoga instructor is doing modifications, I think it's OK for me to do it.” (Diane, evening program)
Flexible and incorporated their wants/needs<br>The instructor requested input from the participants and then incorporated their suggestions into topics areas, postures, breath practices, or meditations.	“You know, I'd asked her something about, I mean, other people from other classes asked about some pelvic floor issues and stuff, and she brought it, you know, sent out links and things and those were helpful. You always knew that if you asked a question and if she didn't have the answer, she'd be getting you one or a list of a book or something, and you could tell she had a ton of experience herself. So, I can't point to any, you know, one thing, but there were a lot of little things that she, that added up.” (Donna, morning program)<br><br>“I liked the breathwork and bringing in different things to try and she was open, like ‘you don't have to like this and if you don't, that's OK.’ […] I like the fact that she gave you options, like you used the chair, don't use the chair, use blocks, don't use blocks, use a band, don't use a band, like she gave you so many different options.” (Sandra, evening program)

Gentle, compassionate, non-judgemental<br>The instructor's interacting style fit the needs of the participants, with many drawing parallels with the types of classes that they had quit previously. The instructor created an environment wherein the participants felt they were allowed to be themselves, to do what they were capable of with encourage to challenge themselves if they wanted to that day.	"The instructor. Jody, I mean, she's outstanding, she has a personality and a professionalism that encourages involvement and interaction. She's incredibly supportive, gentle." (Barbara, morning program)<br><br>"She really certainly made an effort to make it comfortable. And you know the way she was just very empathetic, understanding. Sympathetic, I guess is a good way to put it. It was more a comforting atmosphere rather than, let's say you know competitive or anything. I described the whole thing actually as a healing journey to me." (Pamela, morning program)
Content that supports them to connect with their body<br>The dose of the program was identified as ideal because it provided enough instruction weekly to develop a connection with the self and long enough to form a connection with their peers and the instructor. In addition, the content and the manner in which the instructor taught the content emphasised listening to and respecting the needs of the body, forming a connection with the body.	"I really loved her approach of listening to your own body. She didn't go around the room correcting positioning of our arms and legs like to what end right. It was all about us feeling, I think it was about us feeling more confident and if you're not able to, you know, do a forward bend the way she wanted it, that was not the goal necessarily. And I thought that was really great." (Nancy, morning program)<br><br>"I did find that it helped me be more in tuned to my body and listened to more signals and it helped lower my blood pressure. I did end up in the hospital, I had a cardiac event, but it did help bring my blood pressure down some days after work, when I would do it. So. Before I had my [cardiac] event, I was having what I thought were anxiety attacks and different symptoms, so it did help me kind of be more attuned and paid more attention to what I was feeling so I went to the emergency room when I needed to and did not just put it all down to being stressed out." (Janet, evening program)
**Value and use of optional program features (qualitative responses related to program engagement rates)**	
At-home videos	
No added value<br>Participants did not feel they had the time or a need to add more yoga to their schedule.	"I probably wasn't that interested because if I'm going twice a week, I don't really feel the need to access it on top of twice a week. I'm busy enough, so it was just more than I was willing to consider, I guess." (Donna, morning program)

	"I did look at a couple in the beginning, but then life kind of got in the way and I just did the classes and other things like I was walking and things like that." (Janet, evening program)
Offering recordings of full classes<br>Participants suggested that offering recordings of the classes would be beneficial for when they were unable to attend. These recordings were preferrable because they were comfortable and used to the instructor's style of teaching while also allowing them to continue on with the progressive nature of the program. In addition, participants noted that they did not want the videos during the program but believed it would be beneficial to have access to videos after the program to continue their practice.	"I think you got into the flow of it [structure of class]. So you know, the [pre-recorded] videos concentrated on a certain thing and I could choose that, but I liked the flow of the classes, you knew more or less what kind of things you were going to do there." (Mary, morning program)<br><br>"Because it was a different person and I find it was a little bit more challenging and it was at when I first started I didn't like it because I found it too challenging at first and Jody I found her class is more she starts you from the bottom right?" (Elizabeth, evening program)
Journals	
Different approaches for different needs<br>Some participants found value using the journals on a regular basis (i.e., after every class) to remember thoughts and class content. While others did not see a need to journal, as they felt they processed their emotions/thoughts better internally. Finally, for some participants, once the instructor stopped prompting participants to complete a journal entry (after week 3), they either forgot about the journal or preferred to not journal so that they could more fully engage in the group discussions.	"At the beginning Jody would say if you want to take out your journals and then we'd all scurry and get our bags and get our journals out and start writing in them, and then I don't remember her saying that towards the last, maybe four or so classes so I'd go home and think oh there's my journal empty again, I didn't do anything. Sometimes I'd go back to write something. I think it's a good thing to have, just because I didn't use it doesn't mean it's not a good thing to have, but that was just me." (Susan, morning program)<br><br>"I guess I have trouble expressing my emotions and even writing it down on a piece of paper. I just keep it inside or I don't know. It's just, I'm not good at writing. I never know what to write about or what to so I guess I mean maybe a structured journal, I don't know." (Brenda, evening program)

Group discussions	
Motivating<br>The group discussions were considered an important component of the program that drove connection with the program and facilitated comfort in the classes.	“One of the reasons why I never took up yoga again and also many other classes that I’ve been to in gyms and things like that, I’ve dropped out because you go there, you do your thing and you go away. You know, there's absolutely no social interaction and maybe some people like that I am somebody who seems who needs that bonding and social interaction, I’ve never been able to make it and that was one of the differences with this group, for me. You know, so it's obviously important for me because I’ve never been able to stick to a group in a gym or anything like that.” (Cynthia, morning program)<br><br>“"I found that I mostly stayed even though I was like kind of eager to go home to bed for work. I thought that they were really like valuable and I feel like I learned like stuff from the other women." (Diane, evening program)
Progressive connection<br>Not an immediate benefit (or connection) but one that grew with continuous discussion and sharing of similar experiences.	“would have these sessions at the end where we could, you know, we all talked about everything and nothing kind of thing. But it became everything in the end because I guess we all developed a connection and we felt quite at ease. And you know, you discover that what you're going through, you're not unique and you know it kind of helped me process a lot of my anxieties.” (Cynthia, morning program)<br><br>“"I just thought it was nice to like connect with people after and to like have that like connection because I think that everyone kind of like comes with from all these like different circumstances and then they come together in this place and like some people are worried about work I know some people worried about their families like some people's parents were sick. So it's kind of like a like a little support and a little check in like community connection... So I do really think that's important.” (Diane evening program)
Struggled to form connection online<br>Participants found it difficult to form the same connection with participants who attended primarily online. This was	“The zoom is much harder because of the fact that you're not there and there is, she always had this in a circle at the end, which is brilliant, you know none of this boardroom type stuff. We pull together as a circle, but then the people online were sort of had some had their backs to them, we all turned around if someone spoke,

attributed to struggles associated with ease of communicating.	but I had to do some of the sessions online and it's not the same. I would really encourage on site as much as possible.” (Barbara, morning pogram)<br><br>“Not so much because again, I was always at home. […] If it would have been like mandatory 12 weeks, you got to show up, I would have done it and maybe the benefit for me would have been I met a few people, forced me to do it. While doing it online, hey, I don't have to do that, right?” (Elizabeth, evening program)
Sense of community<br>For the morning program, the group discussions helped to facilitate a sense of emotional safety, cohesion, and connection that lead participants to see themselves as part of a group, which they were committed to trying to maintain post-program.<br><br>However, for the evening program, this was less prevalent. Participants felt they developed less of a connection because the timing of the classes prompted participants to pack up more quickly to head home instead of connecting post-class.	“Last class I think it was emotional because the girls didn't want to say bye.” (Karen, morning program)<br><br>“We're going to continue meeting when we had our little tea on the last day, we exchanged emails and actually their meeting I think it's next week.” (Deborah, morning program)<br><br>“Because of my work, there are times I just didn't linger. I had to go because I had to fit in an hour of work before bed, and so I was conscious of that. Sometimes people were sitting and talking like after the kind of.” (Kathy, evening program)
Notes.	

Table S2. Instructor qualitative responses pertaining to fidelity for co-created 12-week bi-modal hatha yoga program for adults diagnosed with gynecologic cancer		
	Appropriate/Useful	Challenges and Suggestions
Preparation for Program Delivery		
Training Session	**Adequate preparation:** "I felt quite capable and able after the training. […] It was excellent. It was beneficial for sure to meet the team, to meet you and the other people on the team, so that was important to make that personal contact, go through the materials and understanding what everybody's role was helpful. It was good to have the opportunity to ask any questions of you that I had come up when I went through the manual itself."	
Guidebook	**Well formatted and helpful content:** "I think it was easy to find things, that was handy. It was good to have as a reference and included lots of information. It was well laid out, even the fact that, there was the one page [class structure and timing] that you can just briefly glance like it's just helpful. So each part I think was beneficial. […] I like to have all the information and I did reference it a lot. It was good to have at the beginning and I went back to it over and over again, various different parts for various different reasons. What have I done? What have I not done?"	**Add breath awareness practice:** "I would often bring in some other breath awareness practices. I don't particularly like to muck around with people's breath before I get to know them and I want people to get to know their breath and make sure that they have a comfortable starting point before you go into any kind of regulatory regulation type of practice. I would probably do like three sessions, something like that."<br><br>**Add instructor preparation guidance:** "So how do I center and prepare for the class myself? Think about the language I'm going to use to cultivate my own presence. Get grounded, whatever it is that I need to do in order to instruct."<br><br>**Add class theme/topic suggestions:** "Sometimes I would go in with the theme or what I wanted to teach. I would talk about it as part of that check in piece, like 'today is going be a little bit softer, more restful practice,' 'today is going to be like I got lots of energy

		and I'm going to really be all about strength and power,' 'am I going to give myself kindness?' Those kind of things: confidence and power, safe, welcoming, self-permission, compassion, joy. So to have just a list of these are some of the kind of themes that you might want to work with, because I think it's really important piece of the practice - what is your intention today and it could be an instructor's intention or you could put it out towards the class."<br><br>**Add pelvic floor education:** "Something about pelvic floor stuff. It might be really uncomfortable for people to bring the awareness to that area with a group of people, so it was kind of subtle and I didn't do a lot, but to just get them to know it's OK to go there, you know maybe it's OK to go there again and think about that area of the body or trust in that area in the body or notice that area of the body. […] At one point, they started talking a little bit more about their own experience and so oh, the this idea of a pelvic floor dilator, so then I of course had to go and research that because, I don't know what they're talking about. That might be something that could be useful to include in the program, and I kind of did that, we got into the diaphragm and the relationship between the diaphragm and the pelvic floor, and we kind of explored that a little bit just in two sessions and they all found it quite useful. So that might be useful.
Intake meeting and forms	**Useful and necessary:** "It was useful. I probably wouldn't want to teach without it, just because that's how I do it now. It was overall a very useful process. And the timing [15 mins] was good."	**Adding physical capabilities questions:** "There were some questions that I had that I asked standards: if you had previous yoga experience, if they could get up and down from the floor on their own, if they could do weight bearing on their hands, what their sleep and

		energy levels were like, and what's their biggest concern or challenge that they might have at the moment. So it was just some info because I wanted to get a feel before I went in to teach."
Delivery of Program		
Modifiable protocol	**Variation and instructor autonomy:** "It was lovely about the program. You had these things that you want included, but it doesn't have to be this [exact flows] because how boring would that be. I think you'd lose people because it just be, like, so boring time after time after time after time."	
Dosage	**Able to see changes and make connections:** "That [number of weeks] was excellent. It was really starting to kick in probably the last month. It's a bigger commitment, but it's also when they form more as a group. So those extra weeks were important I think."<br><br>"I think 2 at minimum for sure because just the whole time I've been doing this with people you just don't get the same results in once a week. Anecdotally not research wise, but anecdotally that's what I would say."	**A little extra time for shavasana:** "I would suggest you know another 15 minutes just to make sure the long shavasana. But that's just me and my teaching, I guess, but an hour, I think an hour is good. An hour and a half might be too long, particularly if you were doing it in the evening and it's, you know, it's hard, people only have so much time in their day, right? An hour is a big commitment."
Class size		**Challenging to tailor for 10 people:** "I'm not used to teaching that many. It's normally about 6. I mean, it wasn't challenging. I just felt like I couldn't really attend to people as well as I might have if it was a smaller group. Trying to keep my eyes on everyone, I don't know how teachers keep their eyes on 30 people."
Delivery mode		**Adapting teaching style to be more stationary:** "I would do certain things and then the assistant would come and change the position of the camera. I didn't really feel like I could get up and walk around because

		the people that are at home aren't going see what I'm doing. So that was a little bit weird for me."<br><br>**Formally checking on Zoom participants:** "I was trying to keep the people on the zoom in my in the back of my head. So it might make sense to do a meeting for the people coming in remotely all the time – 'is there anything you would like to tell me that is missing for you about my instruction?' Have some kind of midway conversation, just briefly with them. We could even ask them to stay for a couple of minutes after class and have a brief conversation."
Substitute instructor	**Reassuring and ensures continuity in delivery:** "It was useful to know I had a backup because life happens, right? So I think having that as part of the program, that there is somebody that's familiar with the material and what's going on and the people is important."	
Location and room features	**Convenient:** "It was a really great space actually - the fact that they have all the props there is fantastic."	**Better device for monitoring Zoom participants:** "One of the notes I made was if I had a bigger screen or had I been closer to the TV, I would have probably had a better feel for what was going on with the people coming in by zoom - I did feel like some of the times I couldn't see them."<br><br>**Optional, pre-created music playlist:** "I did have a few participants reference music and I think that's a callback to classic studio classes often have music going in the background. Years ago I did but I just find it's too time consuming. So to have a playlist for a more gentle type section of the class. And when you get into more flow or whatever to have something like that is an option would be good."

Group discussion		**Self-conscious about facilitating:** "I think probably the hardest part for me was the discussion piece, facilitating the discussion. I didn't know if I was doing it right, if they we were getting enough out of it. I felt like I was kind of continually in there and towards the end was sometimes more just them and all about them. But it was often, I was still in in the discussion piece."<br><br>**Using a multidirectional microphone:** "Some more prompts and the technical piece, the microphone because it was awkward trying to facilitate the movement of the microphone around the room and people having to speak into a microphone. So I think that can be off putting for some people because all of a sudden again, they're in the spotlight and I'm speaking on a mic and everybody's going to hear, so it kind of interrupts the flow."<br><br>**The necessity of a peer-leader:** "So obviously it depends on the people, but there was particularly one person, maybe 2, and I don't think it would have gone in the direction that it went in without her; she kind of led a lot of the discussions and she opened up and she was vulnerable and honest and thoughtful and reflective. So if you don't have someone kind of doing that, it might not have gone the way it went."
Impact of Program		
Connecting with their bodies	**Connecting with others and themselves:** "Probably the community social aspect, but I also think the movement piece, the yoga itself, was very impactful for them. Part of it was COVID, people had done yoga before, they were afraid to get back into it or maybe after their treatment. For them, they reiterated over and	

	over towards the end, particularly how safe they felt and how nonjudgmental it was. I don't know that you always find that in studio classes. So for them to have a place where they can kind of transition and get their confidence was really valuable. So I think the movement piece and learning to trust their bodies and listen to their bodies and what they can do and know that it can change overtime. I think it was really important.”	
Enriching experience	**An open and understanding environment:** I think it was enriching for them. They had a high level of commitment because perhaps it was enriching but also too I think, it was very meaningful for them to participate in the research because of their own experience. I think that was useful that they could ask me questions and or we could get into experiences and life. Even my age perhaps played a part because I was close to their age. So it might have been different with it 20 year old yoga teacher that for most of these people that was not at that stage in their life, particularly when you're looking at the life aspects and thriving and that sort of thing. I think that was useful.	
